# Prof. D. W. Yandell’s Address before the Teachers’ Convention

**Published:** 1870-05

**Authors:** 


### Prof. D. W. Yandell's Address before the Teachers' Convention.

This address was made in discussing the following proposition, which was the first one adopted by the Convention at Cincinnati in 1867.

"1st. That every student applying for matriculation in a Medical College shall be required to show, either by satisfactory certificate, or by direct examination by a committee of the faculty, that he possesses a knowledge of the common English branches of education, including the first series of mathematics, the elements of the natural sciences, and a sufficient knowledge of Latin and Greek to understand the technical terms of the profession; and that the certificate presented, or the result of the examination thus required, be regularly filed as a part of the records of each Medical College."

Our readers will see the fate of the Teachers' Convention, by Observing the proceedings of the American Medical Association.

